# Evaluation of Factors Influencing Antibody Reduction for Development of Antibody Drug Conjugates

**DOI:** 10.18869/acadpub.ibj.21.4.270

**Published:** 2017-07

**Authors:** Meghdad Abdollahpour-Alitappeh, Majid Lotfinia, Sepand Razavi-Vakhshourpour, Saeed Jahandideh, Hamid Najminejad, Koushan Sineh Sepehr, Reza Moazami, Elnaz Shams, Mahdi Habibi-Anbouhi, Mohsen Abolhassani

**Affiliations:** 1 Hybridoma Lab., Department OF Immunology, Pasteur Institute of Iran, Tehran, Iran; 2 Department of Biochemistry, Pasteur Institute of Iran, Tehran, Iran; 3 Department of Immunology, School of Public Health, Tehran University of Medical Sciences, Tehran, Iran; 4 Biotechnology Research Center, Pasteur Institute of Iran, Tehran, Iran; 5 National Cell Bank of Iran, Pasteur Institute of Iran, Tehran, Iran

**Keywords:** Conjugation, Dithiothreitol, Antibody drug conjugate

## Abstract

**Background:**

Reduction/alkylation is one of the leading strategies for the development of antibody drug conjugates (ADCs). Precise control of the reduction process would not only yield a defined number of free thiols per antibody but also result in development of more homogenous conjugates.

**Methods:**

In the present study, we investigated the effect of various dithiothreitol (DTT) concentrations, temperature conditions, and DTT exposure times on antibody reduction. After antibody reduction, the Ellman’s test and SDS-PAGE analysis were used to evaluate free thiols produced and confirm the reduction process, respectively.

**Results:**

DTT concentration seems to be a potential factor in the reduction process. Concentrations of 0.1, 1, 5, 10, 20, 50, and 100 mM DTT at 37°C for 30 minutes resulted in approximately 0.4, 1.2, 5.4, 7, 8, 8, and 8 thiols per antibody, respectively.

**Conclusion:**

Optimized site-specific conjugation can provide better process control and reproducibility for the development of disulfide-based ADCs.

## INTRODUCTION

Antibody-drug conjugates (ADCs), as a new tool in targeted therapy of cancer, are composed of monoclonal antibodies (mAbs) conjugated to highly toxic small molecule agents (payloads) through small chemical linkers[1,2]. ADCs bring together the targeting advantages of mAbs with the cytotoxic potential of small molecule payloads to enhance specific drug delivery in tumor cells, while sparing healthy tissue and/or cells[3-5]. Methods employed to construct ADCs are generally classified into two broad categories: random modification of mAb amino acid residues and highly regioselective modification[6]. Random modifications of mAbs, despite acceptable biological and functional properties, may impair antigen binding and lead to conjugate heterogeneity. In contrast, selective modification methods have the ability to control the location and stoichiometry of conjugation, resulting in significantly improved mAb conjugates[6]. Reduction/alkylation of solvent-exposed disulfides, as a reliable and robust regioselective strategy, leads to the production of free sulfhydryl groups, allowing for conjugation at specific residues using suitable linkers[7-9]. The process typically involves the reduction of interchain disulfides with dithiothreitol (DTT) or Tris(2-carboxyethyl)phosphine, and subsequent modification of the resulting thiols (-SH groups) with thiol-specific maleimide-containing linker-payloads[6]. The exact number of payloads per antibody is determined by the extent of disulfide reduction. Full reduction of all four interchain disulfide bonds yields a homogeneous construct with eight drugs per antibody, while a partial reduction results in a heterogeneous mixture with 0, 2, 4, 6, or 8 drugs per antibody[10].

Given the above, it is not surprising that antibody reduction plays a vital role in the development of disulfide-based ADCs. Optimization of antibody reduction methods can improve a drug-to-antibody ratio (DAR), drug load distribution/location, and ADC functionality. The purpose of this study was to explore the effects of various temperatures, exposure time, and DTT concentration ranges on antibody reduction. Significant differences were found between the numbers of free thiols per antibody under various conditions.

## MATERIALS AND METHODS

### Antibody preparation

Trastuzumab (Herceptin®) was kindly provided as a gift by Aryogen Pharmed (Karaj, Iran). Normally, it is stored in a lyophilized form at 2-4°C. For each experiment, a fresh stock solution was prepared by dissolving the lyophilized protein in a borate buffer containing 25 mM sodium borate, 25 mM NaCl, and 1 mM diethylenetriaminepentaacetic acid (DTPA; Sigma-Aldrich, USA) (pH 8.0). Trastuzumab was purified in successive steps using Sephadex G-25 gel filtration chromatography, concentrated on a Centricon membrane filter (30 kDa cut-off; Millipore, USA) and stored under sterile conditions in frozen aliquots. The purity and concentration of trastuzumab were determined using SDS-PAGE and UV absorption at 280 nm, respectively.

### Trastuzumab reduction concentration course

Trastuzumab (10 mg/mL) in the borate buffer was reduced with different concentrations of DTT (0.1, 1, 10, 20, 50, and 100 mM; Sigma-Aldrich, USA) under an argon atmosphere at 37°C for 30 minutes. Afterwards, the excess DTT was purified away from the reduced antibody using a Sephadex G-25 column equilibrated with PBSD (PBS containing 1 mM DTPA) and concentrated to 2.5 mg/ml using a 30-kDa cut-off Centricon filter[6].

### Trastuzumab reduction time course

Trastuzumab (10 mg/mL) in the borate buffer was reduced with 5 mM DTT at 37°C. At various times (15, 30, 60, 90, and 120 minutes), the samples were removed, and the excess DTT was purified and concentrated to 2.5 mg/ml, as described above.

### Trastuzumab reduction temperature course

Trastuzumab (10 mg/mL) in borate buffer was reduced with 5 mM DTT at different temperature regimes (4°C, 25°C, 37°C, and 56°C). After 30 minutes, the excess DTT was purified and concentrated to 2.5 mg/ml, as mentioned before.

### Quantification of free thiols

The concentration of thiols produced was quantified by using the Ellman’s reagent[6,11]. Briefly, 4 mg of 5,5’-Dithiobis(2-nitrobenzoic acid) (DTNB; Sigma-Aldrich, USA) was dissolved in 1 ml reaction buffer (0.1 M sodium phosphate, pH 8.0, containing 1 mM EDTA). L-cysteine (at concentrations ranging from 0.25 to 1.5 mM Sigma-Aldrich, USA) was used as a standard substance to confirm the functionality of the Ellman’s reagent. The samples and standard substance (25 µl) were added to 250 μl of reaction buffer, followed by the addition of 5 μl DTNB solution. The reaction was allowed to proceed in the dark at room temperature for 15 minutes. The amount of -SH groups was determined by evaluating the absorbance at 412 nm using an Epoch Microplate Spectrophotometer (BioTek Instruments, Inc., Winooski, VT, USA). The concentration of free sulfhydryls was calculated for each reduction step according to the following equation:

*c*=*A*/*b*E

where *A*=absorbance, *b*=path length in centimeters, *c*=concentration in moles/liter (=M), and E=extinction coefficient.

### Alkylation

To quench the free thiols produced, a 20-fold excess of maleimide over DTT was added to the reduced mAb in the dark at 4°C. Briefly, after incubation periods, aliquots of the reaction were removed, and the reaction was immediately terminated by the addition of 0.1 mL thiol-specific maleimide linkers. Subsequently, the excess maleimide was purified away from the alkylated antibody using a Sephadex G-25 column equilibrated with PBS. Lastly, the Ellman’s test was performed to confirm thiol alkylation.

### SDS-PAGE

The conjugated antibodies were further analyzed by SDS-PAGE, as described by *Laemmli*[12], on a 12% polyacrylamide gel (BioRad, USA) under reducing and non-reducing conditions. The gel was stained with Coomassie blue.

### Statistical analysis

All statistical analyses were conducted using GraphPad Prism 6 (GraphPad Software Inc., CA, USA). Results were presented as the mean±standard deviation of three samples. One-way ANOVA was used to assign significance between the groups. *P* values less than 0.05 were considered to be statistically significant.

## RESULTS AND DISCUSSION

In the present study, we assessed three parameters, including DTT concentrations, temperature conditions, and DTT exposure times for antibody reduction. Four interchain disulfides of trastuzumab, as an IgG1 mAb, were found to have relative susceptibilities to DTT reduction under the various conditions. DTT reduction of trastuzumab yielded approximately zero to eight free thiols per mAb.

In ADCs, a drug can be attached to a mAb through a variety of approaches, including lysine, aldehyde, or cysteine chemistries[10]. Conjugation through antibody cysteines minimizes ADC heterogeneity due to the presence of fewer potential conjugation sites[11,13]. The process typically involves partial reduction of antibody interchain, but not intrachain, disulfide bonds to generate up to eight reactive cysteine thiols, followed by conjugation with payloads containing thiol-specific maleimide linkers[4].

**Fig. 1:**
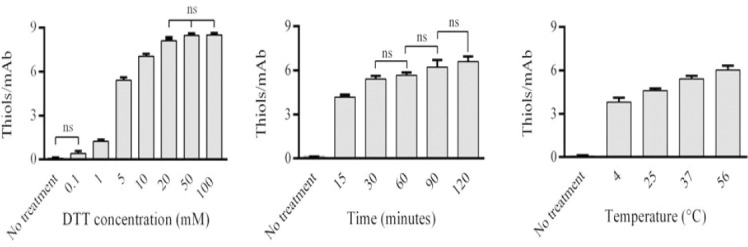
Reduction of trastuzumab under different conditions. Trastuzumab was reduced with (A) various concentrations of DTT, (B) for different time periods, and (C) under different temperature conditions. The statistical analysis showed that (A) DTT concentrations, except for 0.1 mM, (B) time periods, and (C) temperature conditions exhibit significant differences when compared to the “no treatment” group (*P*<0.0001). ns, non-significant

In the first step, trastuzumab was treated with various concentrations of DTT, followed by quantification of free thiol groups using the Ellman’s test. Results showed that concentrations of 0.1, 1, 5, 10, 20, 50, and 100 mM DTT led to approximately 0.4, 1.2, 5.4, 7, 8, 8, and 8 thiols per mAb, respectively. The data, given in Figure 1A, indicated that the number of free thiols levels off at eight; no matter how much the DTT concentration is increased. Consistent with our study, Willner *et al*.[7] demonstrated that the reduction of a chimeric antibody, with up to 100 molar equivalents of DTT, generates only eight sulfhydryls per antibody corresponding to the reduction of the four interchain disulfide bonds.

In the second step, trastuzumab was treated with DTT at various time periods. As shown in Figure 1B, results from the Ellman’s test showed that the 15-, 30-, 60-, 90-, and 120-minute reduction yielded approximately 4.2, 5.4, 5.7, 6.2, and 6.6 thiols per mAb, respectively. A slight difference was found between various time courses. No significant differences were found between 30- and 60-, 60- and 90-, as well as 90- and 120-minute reduction time courses. However, significant differences were observed between the above-mentioned time courses when compared to the 15-minute time course and untreated mAb.

In the third step, trastuzumab was treated with DTT at various temperature conditions. As depicted in Figure 1C, there were significant differences in reduction patterns between various temperature conditions. Results from the Ellman’s test showed that reduction in 4°C, 25°C, 37°C, and 56°C yielded approximately 3.8, 4.6, 5.4 and 6 thiols per mAb, respectively. A slight increase in free thiols per mAb was observed when the samples were incubated at higher temperatures. Reduction in 56°C resulted in more free thiols per antibody, when compared to the other temperatures. Higher DTT concentrations and higher temperatures led to increased antibody reduction, while increased time exposure had the minimum effect on antibody reduction. However, the number of SH equivalents per mole of IgG did not exceed eight at DTT concentrations over 20 mM.

**Fig. 2:**
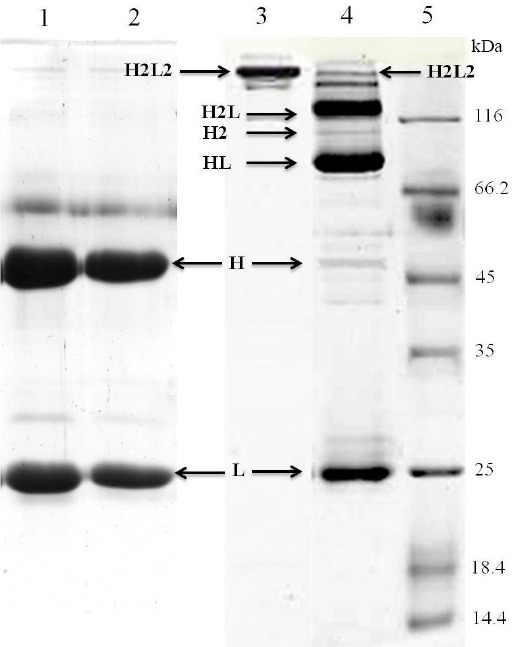
Confirmation of antibody reduction. After reduction of trastuzumab using 5 mM DTT at 37°C for 30 minutes and subsequent alkylation with maleimide-containing drugs, the reduced/alkylated trastuzumab, as well as unreduced trastuzumab with intact interchain as a control, was analyzed on a 12% SDS-PAGE under reducing and non-reducing conditions. Lanes 1 and 2, unreduced trastuzumab and reduced/alkylated trastuzumab, respectively, in the reducing condition; Lanes 3, unreduced trastuzumab under non-reducing conditions; lane 4, reduced/alkylated trastuzumab under non-reducing conditions; lane 5, molecular weight markers (kDa).

After reduction, the free thiols were alkylated with maleimide-containing drugs to cap free cysteine residues. Results from the Ellman’s test indicated no free thiols after alkylation, verifying the correct alkylation process. Following alkylation, the fractions were analyzed on SDS-PAGE gels (Fig. 2). Different species were observed on the gel in reducing (50 and 25 kDa) and non-reducing (150, 125, 100, 75, 50, and 25 kDa) conditions. As shown in Figure 2 (lanes 1 and 2), two separate bands for heavy (H) chain at 50 kDa and light (L) chain at 25 kDa were observed under reducing conditions for both unreduced and reduced/alkylated IgGs, suggesting that the integrity of the reduced/alkylated antibody remained intact. Under non-reducing SDS-PAGE conditions, unreduced trastuzumab with intact interchain disulfide bonds displayed a main band at ~150 kDa that corresponds to the H2L2 form (Fig. 2, lane 3), as expected. As shown in Figure 2 (lane 4), reduced/alkylated trastuzumab exhibited separate bands of molecular weight around 150, 125, 100, 75, 50, and 25 kDa, which fit to H2L2 and H2L, H2, HL, H, and L, respectively. An IgG1 possesses a total of 16 disulfide bonds, including four interchain (2 H-H and 2 H-L) and 12 intrachain disulfide bonds. The four interchain disulfide bonds, clustered in the highly-flexible hinge region, are accessible to reduction without unfolding the antibody[10,14]. Reduction with DTT breaks interchain disulfide bonds and generates cysteines (containing free thiol groups) ranging from zero to eight, leaving the intrachain disulfides intact[6,11,15]. The behavior of the protein in Figure 2 (lane 4) is entirely accounted for the reduction of the two inter-HL disulfides and the two inter-HH disulfides of trastuzumab. Indeed, because some of the interchain disulfide bridges are disrupted by the alkylation, the structure of the typical heterodimeric mAb (H2L2) is no longer maintained in the presence of SDS.

Experiments with purified DAR species have demonstrated that higher DAR ADCs not only are more toxic than lower DAR ADCs at equivalent doses of antibody but also lead to accelerated plasma clearance[10]. In 2004, Hamblett *et al*.[10] revealed that ADC potency *in vitro* directly depends on drug loading (IC50 values 8<4<2 drugs per mAb). They also showed that the *in vivo* antitumor activity of ADCs containing four drugs per mAb was comparable to that containing eight drugs per mAb at equal mAb doses. Importantly, ADCs containing eight drugs per mAb were demonstrated to clear three and fivefold faster than those containing four and two drugs per mAb, respectively. Therefore, in this case, it is necessary to apply lower concentrations of reducing agents for marked reduction of mAbs, which results in development of ADCs with lower DAR species. Considering the findings of the study, it can be deduced that DTT at concentrations of approximately 1.5, 3.5, 7, and 20 mM at 37°C for 30 minutes is suitable to produce ADCs with 2, 4, 6, and 8 drug molecules per mAb, respectively. Sun *et al*.[6] showed that approximately 3.25 and 2.75 molar equivalents of the reducing agents DTT and Tris(2-carboxyethyl) phosphine, respectively, were sufficient to cleave two interchain disulfide bonds, which is consistent with our findings. DTT concentrations, accompanied by temperature conditions, appear to play a more important role in the reduction process, as compared to DTT exposure times. The maximal effects of DTT were observed when its final concentrations were 20 mM at 56°C for 30 minutes. Denaturing conditions at higher temperatures can explain the increased reduction of disulfide bonds at such temperatures.

In conclusion, the present study underscored the important role of the controllable factors in disulfide bond reduction for the development of disulfide-based ADCs. The ability to control the molecular composition in ADCs allows for the generation of well-defined ADCs with potent biological activities. The reduction pattern described herein can help develop conjugates with defined payloads.
